# RETRACTION: Gastroprotective Effects of Plants Extracts on Gastric Mucosal Injury in Experimental Sprague‐Dawley Rats

**DOI:** 10.1155/bmri/9834940

**Published:** 2026-04-24

**Authors:** BioMed Research International

RETRACTION: J. U. Park, J. H. Kang, M. A. A. Rahman, A. Hussain, J. S. Cho, Y. I. Lee, “Gastroprotective Effects of Plants Extracts on Gastric Mucosal Injury in Experimental Sprague‐Dawley Rats,” *BioMed Research International*, 2019, 8759708, https://doi.org/10.1155/2019/8759708

The above article, published online on 17 February 2019 in Wiley Online Library (wileyonlinelibrary.com), has been retracted by agreement the authors; the journal Editor‐in‐Chief, Yoel Klug; and John Wiley & Sons Ltd. The retraction has been agreed due to errors identified by the authors in the contents of the ellagic acid, geniposide and catechine‐7‐O‐*β*‐D‐apiofuranoside stated. These errors cause the results to be considered unreliable and it is therefore retracted with the agreement of the Chief Editor.

